# *Averrhoa carambola* L., *Cyphomandra betacea*, *Myrciaria dubia* as a Source of Bioactive Compounds of Antioxidant Properties

**DOI:** 10.3390/foods12040753

**Published:** 2023-02-09

**Authors:** Dariusz Nowak, Michał Gośliński, Krzysztof Przygoński, Elżbieta Wojtowicz

**Affiliations:** 1 Department of Nutrition and Dietetics, Faculty of Health Sciences, Ludwik Rydygier Collegium Medicum in Bydgoszcz, Nicolaus Copernicus University in Toruń, 87-100 Toruń, Poland; 2 Department of Food Concentrates and Starch Products, Prof. Wacław Dąbrowski Institute of Agricultural and Food Biotechnology, 61-361 Poznań, Poland

**Keywords:** star fruit, tamarillo, camu-camu, polyphenols, antioxidant capacity, bioactive compounds

## Abstract

Natural bioactive compounds play an important role in the prevention of various diseases. The exotic fruits *Averrhoa carambola* L. (star fruit), *Cyphomandra betacea* (tamarillo) and *Myrciaria dubia* (camu-camu) can be valuable sources of phytochemicals with antioxidant properties. The aim of this study has been to compare the antioxidant properties of these exotic fruits, the structure of polyphenolic compounds and the content of vitamin C and β-carotene. All the juices were analyzed for their antioxidant capacity (DPPH and ABTS assays) and the composition of phenolic compounds (TP and FBBB assays, total flavonoid content, total anthocyanins). In addition, HPLC assays were performed to analyse the content of phenolic acids, flavonoids, vitamin C and β-carotene. The results demonstrated that juice from the *Myrciaria dubia* fruit had the highest antioxidant capacity, which was 4.5-fold higher than that of juice from *Averrhola carambola* L., and nearly 7-fold higher than the antioxidant capacity of *Cyphomandra betacea* fruit juice. Additionally, juice from the camu-camu fruit had a 3- to 4-fold higher total polyphenol content (8290 ± 254 mg GAE L^−1^) and a high level of vitamin C (8410.8 ± 16.9 mg AA kg^−1^). In turn, tamarillo juice had a high content of total anthocyanins (5796 mg CGE L^−1^) and phenolic acids (mostly chlorogenic acid and caffeic acid). Juice produced from carambola had a high content of total flavonoids (1345 mg CAE L^−1^), and the composition of these compounds was dominated by flavanols (epicatechin). The research results justify the conclusion that fruits of *Myrciaria dubia*, *Averrhoa carambola* L., *Cyphomandra betacea* are rich sources of bioactive compounds with antioxidant properties, and in the near future may serve as healthful food ingredients.

## 1. Introduction

*Averrhoa carambola* L., known as the star fruit, carambola or five fingers, belongs to the family of *Oxalidaceae*, and originates from South-East Asia. The fruit is also popular in Brazil, Ghana, Guyana and French Polynesia. The star fruit is of high commercial value and is extensively distributed and cultivated in southern China, Southeast Asia, India, and northern South America [1]. In China alone, the annual production of this fruit is two million tons, and the total consumption is about 2.6 million ton per year [2]. The fruit grows on small trees; they are about 7–15 centimetres in length, and up to 9 cm in width. Immature fruit is green, but turn to yellow or yellow-orange when they are ripe. In a cross section, a carambola fruit resembles a star [3,4]. It is frequently used in the preparation of fruit salads, cocktails, juices or functional beverages, jellies, pickles, ice creams, preserves, and sweets, especially in tropical regions [1,5]. Carambola fruit is low in calories (34 kcal/100 g), containing 9.38 g carbohydrates and small amounts of proteins and fat, 0.38 and 0.08 g, respectively [4]. The star fruit is a good source of potassium (167–168 mg/100 g) and such minerals as phosphorus, magnesium and calcium: ca 18, 12 and 6 mg/100 g, respectively [6]. It also contains vitamins C, pro-vitamin A as well as vitamins B_1_, and B_2_, in addition to acids like oxaloacetic, citric, malic, fumaric, tartaric succinic acids, as well as flavonoids [4,6]. These compounds demonstrate antioxidant properties. 

*Cyphomandra betacea* (the tamarillo), also known as the tree tomato, is a representative of the family Solanaceae native to South America, but nowadays it is also cultivated in the United States of America, Australia, New Zealand, Kenya or Portugal. The fruit is oval in shape, 5 to 10 cm in length and 4 to 5 cm in width, with smooth peel, most often red or purple in colour, although yellow and orange fruits are possible [7]. The colour of a fruit depends on the presence of anthocyanins, chlorophylls and carotenoids [8]. The fruit is eaten fresh, cooked in stews and sauces, prepared as chutney and pickles, as well as directly consumed with salads [9]. The composition of the tamarillo fruit is varied, depending on a variety, environmental conditions, ripeness and even part of the fruit. Tamarillo fruit contains 4.89–9.58% of protein, 28.1–52.0% of total sugars [10] and small amounts of lipids 0.1–0.7% [8]. This fruit is rich in potassium (1868–3211 mg/100 g of dry matter), but also contains phosphorus (56–226 mg/100 g), magnesium (48–230 mg/100 g), calcium (16–80 mg/100 g), as well as copper (0.1–3.3 mg/100 g), iron (0.7–2.4 mg/100 g) and zinc (0.2–1.4 mg/100 g) [8]. The tamarillo fruit is a good source of vitamin C, and contains pro-vitamin A and vitamins E, B_1_, B_2_. In its composition, the tamarillo also has organic acids, mainly citric acid (4.0–7.5 g/100 g of dry matter) and a smaller concentration of malic acid (0.3–1.0 g/100 g of dry matter), which–together with sugars (glucose, fructose, and sucrose)–create the characteristic taste of the fruit [10].

Camu-camu (*Myrciaria dubia* (H.B.K.) McVaugh) is an exotic, tropical fruit originating from the Amazon rainforest. The camu-camu shrub grows to a height of 1 to 3 m, and bears round fruits with the diameter of 1.0–3.2 cm, which have a thin, red or purple peel and pink, juicy flesh with one to four seeds inside. Because of its sour and bitter taste, camu-camu berries are most often consumed in a processed form; that is as juices, purees or pulp [11]. The camu-camu fruit is considered to be one of the richest natural sources of vitamin C globally. The content of this vitamin reaches up to 1150 mg/100 g [12], although some genotypes may have as much as 3500 mg/100 g of vitamin C [13]. The *Myrciaria dubia* berries contain small amounts of protein (0.71 g/100 g) and carbohydrates (8.6 g/100 g) [12]. They are a good source of dietary fibre (0.71 g/100 g) and such minerals as potassium, iron and calcium [14]. 

Fruits of *Averrhoa carambola* L., *Cyphomandra betacea*, and *Myrciaria dubia,* besides providing nutrients, are also a source of phytocompounds, such as polyphenols, carotenoids or vitamin C, which shape antioxidant properties. Currently, there are many studies on the natural bioactive compounds that can play an important role in the prevention of various diseases. Plant polyphenols in human diet are beneficial to health as their consumption decreases the incidence of cardiovascular diseases, diabetes, and cancers [15,16] and produce antimicrobial effects [17,18]. However, there has been little research on the phenolic compounds and antioxidant properties of *Averrhoa carambola* L., *Cyphomandra betacea*, and *Myrciaria dubia* fruit. Therefore, the purpose of this study has been to compare the antioxidant properties of these exotic fruits, to identify the structure of polyphenolic compounds, and to determine the content of vitamin C and β-carotene. 

## 2. Materials and Methods

### 2.1. Materials

*Averrhoa carambola* L. (star fruit), *Cyphomandra betacea* (red tamarillo), *Myrciaria dubia* (H.B.K.) McVaugh (camu-camu) were ripe and purchased from a local health food store. Star fruit came from Malaysia, while tamarillo and camu-camu fruit were imported from South America. All the juices were cold pressed from whole fresh and washed fruits. The juices were naturally turbid and free from any additives. The pH values of the juices were measured at room temperature, using a portable pH-meter (Hanna Instruments, Olsztyn, Poland). The juices were stored in sterilized glass bottles at 4 °C until further treatments.

### 2.2. Antioxidant Capacity 

#### 2.2.1. DPPH Assay

The antioxidant capacity of the fruit juices was determined by a modified Yen and Chen method, using 0.1 mmol/L methanol solution of 1,1-diphenyl-2-picrylhydrazyl (DPPH, Sigma-Aldrich, St. Louis, MO, USA) [19]. An amount of 0.1 mL of a sample was added to 2.9 mL of DPPH solution and mixed. The absorbance was measured on a Rayleigh UV-1800 V/VIS spectrophotometer at 517 nm after 30 min of incubation at room temperature in the dark. For each juice, samples were analyzed in three replicates and the results were used to calculate an average value. The percentage of DPPH scavenging was calculated using the equation:

% scavenging = [(A_DPPH_ − A_juice_)/A_DPPH_] × 100

where A_DPPH_ is the absorbance of the DPPH blank solution and A_juice_ is the absorbance of the sample solution.

The obtained value was then substituted into the equation of a previously prepared 6-hydroxy-2,5,7,8-tetramethylchromane-2-carboxylic acid (Trolox-Sigma-Aldrich) calibration curve. The antioxidant capacity of the samples was expressed as milligrams of Trolox equivalents (Sigma-Aldrich) per litre of sample (mg Tx L^−1^).

#### 2.2.2. ABTS Assay

The antioxidant capacity was determined using the Re et al. [20] method with small modifications. In the ABTS method, 2,2′-azinobis-(3-ethyl-benzothiazoline-6-sulfonic acid) diammonium salt (ABTS, Sigma-Aldrich, St. Louis, MO, USA) and potassium persulfate solutions were mixed and stored overnight at room temperature in the dark for 12–16 h. ABTS solution was diluted with methanol to an absorbance of 0.70 ± 0.02 at 734 nm. After addition of 1.0 mL of diluted ABTS solution (A734 nm = 0.700 ± 0.020) to 0.01 mL of antioxidant compounds or Trolox standards in methanol, the absorbance was measured on a Rayleigh UV-1800 V/VIS spectrophotometer at 734 nm against methanol after 1 min. Quantification was performed using a Trolox standard curve. The antioxidant capacity of the samples was expressed as milligrams of Trolox equivalents (Sigma-Aldrich, St. Louis, MO, USA) per litre of sample (mg Tx L^−1^).

### 2.3. Phenolic Compounds

#### 2.3.1. Total Polyphenol Content

The total polyphenol content (TP) of the samples was determined in the Folin–Ciocalteu assay (Sigma-Aldrich) [21]. First, 0.3 mL of a sample was placed in a 10-mL capacity tube; next, 0.05 mL 2 mol/L Folin–Ciocalteu reagent (Sigma-Aldrich, St. Louis, MO, USA) and 0.5 mL 20% sodium carbonate solution were added. The mixture was diluted by addition of 4.15 mL distilled water and mixed. The absorbance was measured on a Rayleigh UV-1800 V/VIS spectrophotometer at 765 nm after 30 min incubation in the dark at room temperature. A calibration curve was performed with gallic acid. The results were expressed as milligrams of gallic acid equivalents per litre of sample (mg GAE L^−1^).

#### 2.3.2. Fast Blue BB Assay 

The phenolic compounds of the samples were also determined with the Fast Blue BB method described by Medina [22] using FBBB reagent (4-benzoylamino-2,5-diethoxybenzenediazonium chloride hemi (zinc chloride) salt; Sigma-Aldrich, St. Louis, MO, USA) in an alkaline medium. An 0.2 mL aliquot of 0.1% Fast Blue BB reagent was added to 2 mL of samples and mixed for 1 min and 0.2 mL 5% sodium hydroxide was added. The absorbance was measured on a Rayleigh UV-1800 V/VIS spectrophotometer at 420 nm after 90 min of incubation in the dark at room temperature. The results are expressed as gallic acid equivalents per litre of sample (mg GAE L^−1^).

### 2.4. Total Flavonoid Content

The total flavonoid content was measured using the colorimetric assay developed by Kapci et al. [23]. Briefly, 0.3 mL of 5% sodium nitrite was added to 1 mL of the sample at zero time. After 5 min, 0.3 mL of 10% aluminium chloride was added. At the 6th min, 2 mL of 1M sodium hydroxide was added. The mixture was diluted by addition of 2.4 mL distilled water and mixed. The absorbance was measured on a Rayleigh UV-1800 V/VIS spectrophotometer at 510 nm. The total flavonoids content was determined using a (+)-catechin (Sigma-Aldrich, St. Louis, MO, USA) standard curve and was expressed as milligrams of catechin equivalents per litre of sample (mg CAE L^−1^).

### 2.5. Total Anthocyanins

Total anthocyanins (TA) were determined using the pH differential method (AOAC Official Method 2005.02) [24]. Juices were diluted according to appropriate dilution ratios (one part sample and four parts buffer) by adding both 0.025 mol L^−1^ KCl (pH 1.0) or 0.4 mol L^−1^ CH_3_COONa·3H_2_O (pH 4.5) buffer solutions (Avantor Performance Materials, Gliwice, Poland). Samples were mixed and left in the dark for 30 min. Absorbance was measured on a Rayleigh UV-1800 V/VIS spectrophotometer at 520 nm and 700 nm, and the results were calculated using the following formula:

TA = A × MW × DF × 10^3^ × (E × 1)^−1^

where:

A = [(A_520_ − A_700_)_pH1.0_ − (A_520_ − A_700_)_pH4.5_], where A_520_ is the absorbance measured at 520 nm and A_700_ is the absorbance measured at 700 nm, at pH 1.0 and 4.5, respectively;

MW (molecular weight) = 449.2 g × mol^−1^ for cyanidin-3-glucoside; DF = dilution factor established in D; 1 = path length in cm; E = 26,900 molecular extinction coefficient in L × mol^−1^ × cm^−1^ for cyd-3-glu; 10^3^ = factor for conversion from g to mg.

Total anthocyanins were expressed as milligrams of cyanidin-3-mono-glucoside equivalents per litre of sample mg CGE L^−1^ juice.

### 2.6. HPLC Analysis of Phenolic Acids and Flavonoids

Phenolic acids and selected flavonoids were determined using HPLC methods described by Krygier et al. [25] and Hertog et al. [26] based on the parameters established in our previous studies [27,28]. The analyses were performed using a Dionex LC system equipped with a photodiode array detector (PAD, Dionex, Sunnyvale, CA, USA) and the absorption spectra were recorded in the range of 200–600 nm. The flow rate was 1 mL/min, the column temperature was 30 °C and the injection volume was 20 μL. Qualitative identification was done by comparing the retention times and spectra with the standards. Simultaneous monitoring was performed at 280 nm for phenolic acids and 360 nm for flavonoids. 

Phenolic acids were determined according to the method described by Krygier et al. [25]. The separation was performed on an Ascentis C18 (Supelco, Sigma-Aldrich, St. Louis, MO, USA) column (250 mm × 4.6 mm; 5 μm). The binary mobile phase consisted of 0.1% (*v*/*v*) formic acid in methanol (eluent A) and methanol-acetonitrile (80:20, *v*/*v*; eluent B).

Flavonoids were determined using a modified Hertog et al. [26] method, after their acidic hydrolysis. The separation was performed on an Ascentis (Supelco) C18 column (250 mm × 4.6 mm; 5 μm). The binary mobile phase consisted of 0.1% (*v*/*v*) formic acid in water-methanol (75:25, *v*/*v*, pH 2.7; eluent A) and 0.1% (*v*/*v*) formic acid in methanol (eluent B). 

### 2.7. HPLC Analysis of Ascorbic Acid and β-Caroten

Ascorbic acid was determined using HPLC methods of AOAC [29] with slight modification. The separation was performed on a Thermo Electron Co., column BetaBasic-18 (150 mm × 4.6 mm, 5 µm). The isocratic mobile phase consisted of 0.1% o-phosphoric acid in water. 

β-carotene was determined using HPLC normalised methods of EN-12823-2:2000 [30] with slight modification. The separation was performed on a Kinetex C18 (Phenomenex) column (150 mm × 4.6 mm, 2.6 µm). 

### 2.8. Statistical Analysis

The results were statistically analysed by calculating the mean and standard deviation. The interpretation of the results was performed with MS Excel 2019 Analysis ToolPak software, one-way analysis of variance (ANOVA) using the Tukey’s post-hoc test: different letters in the same row or column in the tables indicated statistical significance (at least *p* < 0.05). 

## 3. Results and Discussion

Antioxidant capacity and phenolic compounds of the tested fruit juices are presented in Table 1. The highest antioxidant capacity was demonstrated by the juice from *Myrciaria dubia* fruits. The antioxidant capacity of the camu-camu fruit juice was 4.5-fold higher than that of the *Averrhola carambola* L. juice, and nearly 7-fold higher than that of *Cyphomandra betacea* juice. The camu-camu juice also had a 3- to 4-fold higher total polyphenol content (TP) (determined with the Folin-Ciocalteu method) than the other analyzed juices. Slightly smaller differences between the analysed juices were manifested in the FBBB method. In turn, the *Averrhoa carambola* L. juice had higher total polyphenols (determined with both methods) than the juice from *Cyphomandra betacea*. This finding was confirmed in another study [31], although the total amounts of polyphenols determined in our experiment were much higher. On the other hand, the total content of polyphenols determined with the F-C method in the tamarillo fruit juice was similar to the values obtained by Vasco et al. [32] for a purple-red variety of this fruit.

It was noted that the results on the total content of polyphenols in the FBBB method were higher than obtained using the Folin-Ciocalteu method, except for the results of camu-camu juice. The Fast Blue BB method usually demonstrates higher values of gallic acid equivalents (GAE) than the Folin–Ciocalteu assay does [22]. In our study, the FBBB method tended to yield results which were 1.2 to 1.3 higher than the ones obtained with the Folin-Ciocalteu method. Even higher results achieved with the FBBB method than with the F-C method were shown by Kang et al. [33], who were the first to compare both methods. Kang et al. concluded that the results of polyphenols using the Folin–Ciocalteu assay may not be accurate because fruits, vegetables and juices contain such ingredients as glucose, fructose, carotenoids or ascorbic acid which may distort the measurements of TP [33]. On the other hand, Roslan et al. showed that total polyphenol content estimated using the Folin-Ciocalteu assay were significantly higher than obtained from the FBBB assay in all the analysed samples (green tea and commercial fruit juices). The authors concluded that the F-C assay was significantly affected by the presence of ascorbic acid compared to the FBBB assay [34]. This conclusion was confirmed in our study.

Furthermore, we determined some correlation between the analytical methods used (Table 2). DPPH and ABTS assays yielded a high correlation coefficient (*R*^2^ = 0.999). In addition, both methods demonstrated a high linear correlation with TP (0.999 and 0.998, respectively), which confirms that polyphenols contributed more to the antioxidant capacity than other compounds. Relatively high correlation coefficients were noted between TP and vitamin C (0.995), and a lower correlation was determined between the FBBB assay and vitamin C (0.910). 

The camu-camu juice had a high content of polyphenols according to the Folin-Ciocalteu method (8290 ± 254 mg GAE L^−1^) and a very high content of ascorbic acid (8410.8 ± 16.9 mg kg^−1^) (Table 3), at a much lower total amount of polyphenols determined with the FBBB method (Table 1). The high content of polyphenols in camu-camu fruits has been emphasised by Akter et al. [14], Rufino et al. [35] and Aguiar and Souza [12], and variations in concentrations of polyphenols arise from differences in the ripeness of fruit, cultivar and ways of preparing the material (pulp, extract). Other authors have also reported a high content of vitamin C in camu-camu fruit pulp (from 800 to 6100 mg AA 100 g^−1^) [13,36]. It has also been found that the camu-camu fruit has a higher vitamin C content than other Brazilian fruits, like acerola (1053 mg 100 g^−1^ fresh matter) and acai (84.0 mg 100 g^−1^ fresh matter) [12,35].

Our study showed that the camu-camu juice had a high vitamin C content, but was not a source of β-carotene, in contrast to the juice from *Cyphomandra betacea*, i.e., tamarillo fruit (Table 3). The results concerning vitamin C and β-carotene in the juices from *Averrhola carambola* L. and tamarillo were lower than reported elsewhere [6,8,10], but this was due to the differences in the analyzed material (juice versus fresh fruit, pulp or extract). Our experiment showed that the tamarillo juice had a high content of total anthocyanins (TA)-5796 mg CGE L^−1^ (Table 1), much higher than in the camu-camu juice-252 mg CGE L^−1^, while the carambola juice contained much of total flavonoids (TF) 1345 mg CAE L^−1^.

Our analysis of the composition of polyphenols (HPLC) showed that the tamarillo (*Cyphomandra betacea*) juice was a rich source of phenolic acids (mainly chlorogenic and caffeic acids), resveratrol (belonging to the stilbenes) and flavonols as well as kaempferol (Table 3). 

Our results are similar to those reported by other researchers, who demonstrated in tamarillo the presence of flavonoids and phenolic acids (mainly caffeic and chlorogenic acids) [37,38,39]. Other researchers have identified other phenolic compounds in the tamarillo fruit or else the identification of such compounds has been rather poor. Espin et al. [40] showed that the New Zealand purple tamarillo fruit was high in hydroxycinnamates (421.6 mg 100 g^−1^ dry pulp). Tentatively identified phenolic acids and derivatives in tamarillo included quinic acid, caffeoyl glucoside, dehydrodiferulic acids, 3- and 5-O-caffeoylquinic acids, feruloyl glucoside, rosmarinic acid glucosides, malonyl derivative of rosmarinic acid glucoside, and rosmarinic acid [8,40]. 

The main flavonoids determined in the *Averrhoa carambola* L. juice were epicatechin and catechin (flavanols), quercetin and myricetin (flavonols), apigenin (flavones), in addition to small amounts of phenolic acids. Luan et al. [1] indicated about 132 compounds in star fruit (fruits, leaves, roots) e.i. flavonoids, terpenes, phenylpropanoids, and their glycosides. The HPLC analyses conducted on *Averrhoa carambola* fruit demonstrated the presence of various phenolic acids and flavonoids such as gallic acid, 4-hydroxycinnamic acid, 4-hydroxy-3-methoxycinnamic acid, vanillic acid, kaempferol, luteolin, naringenin, and quercetin in aqueous and ethanol extracts [41]. Similar to our study, other researchers reported epicatechins (in the fruit) [42] and apigenin (in the leaves) [43].

The camu-camu juice tested in our experiment was found to contain flavones (mainly apigenin) and small quantities of flavonols (mainly kaempferol) as well as phenolic acids. In other studies, the camu-camu fruits were determined to have various polyphenols [14,44,45,46]. Chirinos et al. [44] showed 30 different phenolic compounds, which were detected with the HPLC-PAD method. Same as in our research, they detected the presence of flavonols, flavanols and phenolic acids. Furthermore, researchers showed, that the camu-camu fruit contains flavanones (naringenin, eriodictyol) [14] and anthocyanins, ellagitannins and condensed tannins [46]. The difference in the content of phenolic compounds resulted from fruit maturity, morphological part of the plant (leaves, mesocarp, seeds), a different extraction method and the solvent mixture.

Our research yielded quite detailed determinations of the concentrations of major bioactive compounds with antioxidant properties in juices from ripe fruits of *Averrhoa carambola* L., *Cyphomandra betacea* and *Myrciaria dubia.* In this context, noteworthy is the observation that as camu-camu fruits were maturing, the content of vitamin C decreased while the levels of anthocyanins, flavonols and flavonoids increased alongside the growing antioxidant capacity measured with the DPPH assays [11,44]. 

Owing to their high content of phytocompounds, camu-camu fruits can be classified as a superfood, which is worth further investigation in order to take advantage of their properties in the prophylaxis of non-infectious illnesses. Other researcher also acknowledged the health aspect of the above fruits. Lakmal et al. [5] showed the beneficial effect of star fruit (*Averrhoa carambola*), associated with the presence of various phytochemicals with antioxidant properties. Moreover, tamarillo fruit extracts have a beneficial effect associated with the presence of numerous phytochemicals [8,47]. Moreover, Santos et al. [48] pointed out that it is not only the camu-camu pulp and juice but also camu-camu seeds and peels that can be sources of bioactive products. Fruit phenolics are important dietary antioxidant and anti-inflammatory constituents [45]. Recently, the impact of fruit extracts has been verified on specific cell lines. For example, the optimised camu-camu extract displayed a cytotoxic effect and antiproliferative activity against cancer cells [46]. Further studies concerning the potential health benefits of exotic fruit juices and extracts should be continued.

## 4. Conclusions

The research results presented in this article have revealed that the high antioxidant capacity of camu-camu juice arose mainly from the high content of vitamin C in the plant’s fruit, which also resulted in the high content of polyphenols determined with the Folin-Ciocalteu method. The research results presented in this article have revealed the highest antioxidant properties of camu-camu juice, which had a very high vitamin C content and total polyphenol content, regardless of the analytical method used. Polyphenols were the key compounds in the composition of juices from *Averrhoa carambola* L. and *Cyphomandra betacea* affecting their antioxidant capacity. The *Cyphomandra juice* was a rich source of anthocyanins and phenolic acids (mostly chlorogenic acid and caffeic acid), as well as flavonols and resveratrol. 

Star fruit juice had a higher total polyphenol content (in both methods) and total flavonoid content than tamarillo juice, which was a rich source of anthocyanins and phenolic acids (mostly caffeic and chlorogenic acid), as well as resveratrol. Furthermore, the flavonoids determined in the *Averrhoa carambola* L. juice contained mostly flavanols (mainly epicatechin) and, in smaller amounts, flavonols (mainly quercetin) and flavones (apigenin). This juice, same as the juice from camu-camu fruits, did not contain resveratrol and was a less abundant source of phenolic acids, in contrast to tamarillo juice. This article gives a detailed presentation of the structure of phenolic acids, which enabled us to compare these three, exotic juices. Without doubt, the analyzed juices, from the fruits of *Myrciaria dubia*, *Averrhoa carambola* L., *Cyphomandra betacea*, were rich in bioactive compounds with antioxidant properties, and may not only become an alternative to juices from local fruits but could also be used in preventive healthcare.

## Figures and Tables

**Table 1:** Antioxidant capacity and polyphenols of fruit juices.

	*Averrhoa* *carambola* L. (Star Fruit)	*Cyphomandra* *betacea * (Tamarillo)	*Myrciaria* *dubia * (Camu Camu)
pH	3.5 ± 0.1 ^a^	3.5 ± 0.1 ^a^	3.0 ± 0.1 ^b^
DPPH [mg Tx L^−1^]	1268 ± 80 ^b^	858 ± 15 ^c^	5763 ± 247 ^a^
ABTS [mg Tx L^−1^]	1906 ± 146 ^b^	1214 ± 27 ^c^	6981 ± 349 ^a^
TP [mg GAE L^−1^]	2464 ± 153 ^b^	1957 ± 187 ^b^	8290 ± 254 ^a^
FBBB [mg GAE L^−1^]	3356 ± 87 ^b^	2400 ± 63 ^c^	4799 ± 24 ^a^
TF [mg CAE L^−1^]	1345 ± 17 ^a^	206 ± 15 ^b^	tr.
TA (mg CGE L^−1^)	tr.	5796 ± 72 ^a^	252 ± 10 ^b^

Abbreviations: TP, total polyphenol content; FBBB, Fast Blue BB reagent; TF, total flavonoid content; TA, total anthocyanins; tr.–trace (LOD = 5 mg L^−1^). Data are mean ± standard deviation (n = 3). Statistical analysis was performed by one-way ANOVA using the Tukey’s post hoc test: different letters in the same row indicate statistical significance (*p* ≤ 0.05).

**Table 2:** The correlation coefficients (*R*^2^).

	DPPH	ABTS	TP	FBBB	TF	TA	Vit. C	β-Carotene
DPPH		0.999	0.999	0.945	−0.557	−0.532	0.995	−0.599
ABTS	0.999		0.998	0.955	−0.528	−0.560	0.992	−0.626
TP	0.999	0.998		0.942	−0.560	−0.527	0.995	−0.594
FBBB	0.945	0.955	0.942		−0.256	−0.778	0.910	−0.826
TF	−0.557	−0.528	−0.560	−0.256		−0.407	−0.633	−0.332
TA	−0.532	−0.560	−0.527	−0.778	−0.407		−0.449	0.996
Vit. C	0.995	0.992	0.995	0.910	−0.633	−0.449		−0.520
β-carotene	−0.599	−0.626	−0.594	−0.826	−0.332	0.996	−0.520	

**Table 3:** HPLC analysis of selected phenolic compounds and vitamins (mg kg^−1^ ± SD).

Compounds	*Averrhoa * *carambola* L. (Star Fruit)	*Cyphomandra* *betacea* (Tamarillo)	*Myrciaria* *dubia* (Camu Camu)
Free phenolic acids			
Gallic acid	nd	nd	nd
Chlorogenic acid	nd	24.1 ± 0.2	nd
Caffeic acid	nd	2.6 ± 0.1	tr.
Coumaric acid	nd	nd	nd
Ferulic acid	0.2 ± 0.1	nd	nd
Bound phenolic acids			
Gallic acid	nd	1.5 ± 0.1 ^a^	0.7 ± 0.1 ^b^
Caffeic acid	nd	80.6 ± 0.6	tr.
Coumaric acid	6.0 ± 0.1 ^a^	3.8 ± 0.1 ^b^	0.2 ± 0.1 ^c^
Ferulic acid	2.7 ± 0.1 ^b^	11.8 ± 0.1 ^a^	tr.
Flavanols			
Catechin	1.4 ± 0.1	tr.	tr.
Epicatechin	12.1 ± 0.1	nd	nd
Flavonols			
Myricetin	0.4 ± 0.1	nd	nd
Quercetin	2.3 ± 0.2	tr.	nd
Kaempferol	tr.	2.3 ± 0.2 ^a^	0.4 ± 0.1 ^b^
Flavones			
Luteolin	nd	nd	nd
Apigenin	2.6 ± 0.1 ^a^	tr.	1.8 ± 0.4 ^b^
Stilbenes			
Resveratrol	nd	24.9 ± 0.4	nd
Vitamins			
Ascorbic acid	36.0 ± 1.4 ^c^	220.7 ± 0.7 ^b^	8410.8 ± 16.9 ^a^
β-carotene	0.13 ± 0.01 ^b^	2.52 ± 0.03 ^a^	tr.

Bound phenolic acids = phenolic acids after its alkaline hydrolysis; Phenolic acids, Flavanols, Flavones, Resveratrol LOD = 0.1 mg/kg; Vitamin: ascorbic acid LOD = 0.5 mg/kg; β-carotene LOD = 0.004 mg/kg; nd–not detected, tr.–trace. Statistical analysis was performed using one-way ANOVA using the Tukey’s *post hoc* test: different letters in the same row indicate statistical significance (*p* ≤ 0.05).

## Data Availability

The data presented in this study are available on request from the corresponding author.

## References

[1] Luan F., Peng L., Lei Z., Jia X., Zou J., Yang Y., He X., Zeng N. (2021). Traditional uses, phytochemical constituents and pharmacological properties of *Averrhoa carambola* L.: A review. Front. Pharmacol..

[2] Wu S.S., Sun W., Xu Z.C., Zhai J.W., Li X.P., Li C.R., Zhang D., Wu X., Shen L., Chen J. (2020). The genome sequence of Star Fruit (*Averrhoa carambola*). Hortic. Res..

[3] Dasgupta P., Chakraborty P., Bala N.N. (2013). *Averrhoa carambola*: An updated review. Int. J. Pharm. Sci. Rev. Res..

[4] Ferrara L. (2018). *Averrhoa carambola* Linn: Is it really a toxic fruit?. Int. J. Med. Rev..

[5] Lakmal K., Yasawardene P., Jayarajah U., Seneviratne S.L. (2021). Nutritional and medicinal properties of Star fruit (*Averrhoa carambola*): A review. Food Sci. Nutr..

[6] Muthu N., Lee S., Phua K., Bhorel S. (2016). Nutritional, medicinal and toxicological attributes of star-fruits (*Averrhoa carambola* L.): A review. J. Bioinform..

[7] Mwithiga G., Mukolwe M.I., Shitanda D., Karanja P.N. (2007). Evaluation of the effect of ripening on the sensory quality and properties of tamarillo (*Cyphomandra betaceae*) fruits. J. Food Eng..

[8] Wang S., Zhu F. (2020). Tamarillo (*Solanum betaceum*): Chemical composition, biological properties, and product innovation. Trends Food Sci. Technol..

[9] Ghosal M., Chhetri P.K., Ghosh M.K., Mandal P. (2013). Changes in antioxidant activity of *Cyphomandra betacea* (Cav.) Sendtn. Fruits during maturation and senescence. Int. J. Food Prop..

[10] Acosta-Quezada P.G., Raigon M.D., Riofrío-Cuenca T., García-Martínez M.D., Plazas M., Burneo J.I., Figueroa J.G., Vilanova S., Prohens J. (2015). Diversity for chemical composition in a collection of different varietal types of tree tomato (*Solanum betaceum* Cav.), an Andean exotic fruit. Food Chem..

[11] Langley P.C., Pergolizzi J.V., Taylor R., Ridgway C. (2015). Antioxidant and associated capacities of Camu camu (*Myrciaria dubia*): A systematic review. J. Altern. Complement. Med..

[12] Aguiar J.P.L., Souza F.C.A. (2016). Camu-Camu super fruit (*Myrciaria dubia* (H.B.K) Mc Vaugh) at different maturity stages. Afr. J. Agric. Res..

[13] Aguiar J.P.L., Souza F.C.A. (2021). Camu-Camu (*Myrciaria dubia*) from the Amazon. Int. J. Adv. Eng. Res. Sci..

[14] Akter M.S., Oh S., Eun J.B., Ahmed M. (2011). Nutritional compositions and health promoting phytochemicals of camu-camu (*Myrciaria dubia*) fruit: A review. Food Res. Int..

[15] Zhang Z., Li X., Sang S., McClements D.J., Chen L., Long J., Jiao A., Jin Z., Qiu C. (2022). Polyphenols as plant-based nutraceuticals: Health effects, encapsulation, nano-delivery, and application. Foods.

[16] Cebulak T., Krochmal-Marczak B., Stryjecka M., Krzysztofik B., Sawicka B., Danilcenko H., Jarienè E. (2023). Phenolic acid content and antioxidant properties of edible potato (*Solanum tuberosum* L.) with various tuber flesh colours. Foods.

[17] Du H., Wu J., Li H., Zhong P.-X., Xu Y.-J., Li C.-H., Wang L.-S. (2013). Polyphenols and triterpenes from Chaenomeles fruits: Chemical analysis and antioxidant activities assessment. Food Chem..

[18] Kranz S., Guellmar A., Olschowsky P., Tonndorf-Martini S., Heyder M., Pfister W., Reise M., Sigusch B. (2020). Antimicrobial effect of natural berry juices on common oral pathogenic bacteria. Antibiotics.

[19] Yen G., Chen H.Y. (1995). Antioxidant activity of various tea extract in relation to their antimutagenicity. J. Agric. Food Chem..

[20] Re R., Pellegrini N., Proteggente A., Pannala A., Yang M., Rice-Evans C. (1999). Antioxidant activity applying an improved ABTS radical cation decolorization assay. Free Radic. Biol. Med..

[21] Singleton V.L., Orthofer R., Lamuela-Raventos R.M. (1999). Analysis of total phenols and other oxidation substrates and antioxidants by means of Folin—Ciocalteu reagent. Methods Enzymol..

[22] Medina M.B. (2011). Determination of the total phenolics in juices and superfruits by a novel chemical method. J. Funct. Foods.

[23] Kapci B., Neradova E., Cizkova H., Voldrich M., Rajchl A., Capanoglu E. (2013). Investigating the antioxidant capacity of chokeberry (*Aronia melanocarpa*) products. J. Food Nutr. Res..

[24] Lee J., Durst R.W., Wrolstad R.E. (2005). Determination of total monomeric anthocyanin pigment content of fruit juices, beverages, natural colorants, and wines by the pH differential method: Collaborative study. J. AOAC Int..

[25] Krygier K., Sosulski F., Hogge L. (1982). Free esterified and insoluble bound phenolic acids. I. Extractions and purification procedure. J. Agric. Food Chem..

[26] Hertog M.G.L., Hollman P.C.H., Venema D.P. (1992). Optimization of quantitative HPLC determination of potentially anticarcinogenic flavonoids in vegetables and fruits. J. Agric. Food Chem..

[27] Nowak D., Gośliński M., Wojtowicz E. (2016). Comparative analysis of the antioxidant capacity of selected fruit juices and nectars: Chokeberry juice as a rich source of polyphenols. Int. J. Food Prop..

[28] Nowak D., Gośliński M., Przygoński K., Wojtowicz E. (2018). The antioxidant properties of exotic fruit juices from acai, maqui berry and noni berries. Eur. Food Res. Technol..

[29] Schimpf K., Thompson L., Baugh S. (2013). Vitamin C in infant formula and adult/pediatric nutritional formula by HPLC with UV detection: First Action 2012.21. J. AOAC Int..

[30] (2000). Foodstuffs—Determination of vitamin A by high performance liquid chromatography—Part 2: Measurment of β-carotene.

[31] Ruiz-Torralba A., Guerra-Hernández E., García-Villanova B. (2018). Antioxidant capacity, polyphenol content and contribution to dietary intake of 52 fruits sold in Spain. CyTA J. Food.

[32] Vasco C., Ruales J., Kamal-Eldin A. (2008). Total phenolic compounds and antioxidant capacities of major fruits from Ecuador. Food Chem..

[33] Kang J., Thakali K.M., Xie C., Kondo M., Tong Y., Ou B., Jensen G., Medina M.B., Schauss A.G., Wu X. (2012). Bioactivities of açaí (*Euterpe precatoria* Mart.) fruit pulp, superior antioxidant and anti-inflammatory properties to *Euterpe oleracea* Mart. Food Chem..

[34] Roslan A., Ando Y., Azlan A., Ismail A. (2019). Effect of glucose and ascorbic acid on total phenolic content estimation of green tea and commercial fruit juices by using Folin-Ciocalteu and Fast Blue BB assays. Pertanika J. Trop. Agric. Sci..

[35] Rufino M.S.M., Alves R.E., Fernandes F.A.N., Brito E.S. (2011). Free radical scavenging behavior of ten exotic tropical fruits extracts. Food Res. Int..

[36] Yuyama K., Aguiar J.P.L., Yuyama L.K.O. (2002). Camu-camu: Um fruto antástico como fonte de vitamina C. Acta Amaz..

[37] Muñoz-Jauregui A.M., Ramos-Escudero F., Alvarado-Ortiz Ureta C., Castañeda Castañeda B., Lizaraso Caparó F. (2009). Evaluación de compuestos con actividad biológica en cáscara de camu camu (*Myrciaria dubia*), guinda (*Prunus serotina*), tomate de árbol (*Cyphomandra betacea*) y carambola (*Averrhoa carambola* L.) cultivadas en Perú. Rev. Soc. Chem. Perú.

[38] Orqueda M.E., Torres S., Zampini I.C., Cattaneo F., Di Pardo A.F., Valle E.M., Jiménez-Aspee F., Schmeda-Hirschmann G., Isla M.I. (2020). Integral use of Argentinean *Solanum betaceum* red fruits as functional food ingredient to prevent metabolic syndrome: Effect of in vitro simulated gastroduodenal digestion. Heliyon.

[39] Reyes-Garcia V., Ttpsaus A., Perez-Chabel L., Juarez Z.N., Cardoso-Ugarte G.A., Perez-Armendariz B. (2021). Exploration of the potential bioactive molecules of tamarillo (*Cyphomandra betacea*): Antioxidant properties and prebiotic index. Appl. Sci..

[40] Espin S., Gonzalez-Manzano S., Taco V., Poveda C., Ayuda-Durán B., Gonzalez-Paramas A.M., Santos-Buelga C. (2016). Phenolic composition and antioxidant capacity of yellow and purple-red Ecuadorian cultivars of tree tomato (*Solanum betaceum* Cav.). Food Chem..

[41] Khanam Z., Sam K.H., Zakaria N.H.B.M., Ching C.H., Bhat I.U.H. (2015). Determination of polyphenolic content, HPLC analyses and DNA cleavage activity of Malaysian *Averrhoa carambola* L. fruit extracts. J. King Saud Univ. Sci..

[42] Gunawardena D.C., Jayasinghe L., Fujimoto Y. (2015). Phytotoxic constituents of the fruits of *Averrhoa carambola*. Chem. Nat. Compd..

[43] Moresco H.H., Queiroz G.S., Pizzolatti M.G., Brighente I.M.C. (2012). Chemical constituents and evaluation of the toxic and antioxidant activities of *Averrhoa carambola* leaves. Rev. Bras. Farmacogn..

[44] Chirinos R., Galarza J., Betalleluz-Pallardel I., Pedreschi R., Campos D. (2010). Antioxidant compounds and antioxidant capacity of Peruvian camu camu (*Myrciara dubia* [H.B.K.] McVaugh) fruit at different maturity stages. Food Chem..

[45] Reynertson K.A., Yang H., Jiang B., Basile M.J., Kennelly E.J. (2008). Quantitative analysis of antiradical phenolic constituents from fourteen edible *Myrtaceae* fruits. Food Chem..

[46] Fidelis M., do Carmo M.A.V., da Cruz T.M., Azevedo L., Myoda T., Furtado M.M., Marques M.B., Sant’Ana A.S., Genovese M.I., Oh W.Y. (2020). Camu-camu seed (*Myrciaria dúbia*)—From side stream to an antioxidant, antihyperglycemic, antiproliferative, antimicrobial, antihemolytic, anti-inflammatory, and antihypertensive ingredient. Food Chem..

[47] Diep T.T., Rush E.C., Yoo M.J.Y. (2022). Tamarillo (*Solanum betaceum* Cav.): A review of physicochemical and bioactive properties and potential applications. Food Rev. Int..

[48] Santos I.L., Miranda L.C.F., da Cruz Rodrigues A.M., da Silva L.H.M., Amante E.R. (2022). Camu-camu [*Myrciaria dubia* (HBK) McVaugh]: A review of properties and proposals of products for integral valorization of raw material. Food Chem..

